# Causes of sickness absenteeism in the adult population of Qatar: A cross-sectional analysis of health records

**DOI:** 10.5339/qmj.2025.70

**Published:** 2025-04-21

**Authors:** Sami Abdeen, Ahmed Sameer Alnuaimi, Muna Abed Alah, Salwan Al-Kubaisi, Iheb Bougmiza

**Affiliations:** 1 Department of Preventative Health, Primary Health Care Corporation (PHCC), Doha, Qatar; 2 Department of Clinical Research, Primary Health Care Corporation (PHCC), Doha, Qatar; 3 Clinical Effectiveness Department, Primary Health Care Corporation (PHCC), Doha, Qatar; 4 Primary Health Care Corporation (PHCC), Doha, Qatar; 5 Department of Community and Preventive Medicine, Primary Health Care Corporation (PHCC), Doha, Qatar; 6 Community Medicine Department, College of Medicine, Sousse University, Sousse, Tunisia; 7 College of Medicine, QU Health, Qatar University, Doha, Qatar *Email: SAbdeen@phcc.gov.qa

**Keywords:** Medical causes, Qatar, sick leave, sickness absenteeism, standardized classification

## Abstract

**Background:**

Sickness absenteeism is a significant global health concern, and understanding its causes is crucial for developing effective prevention strategies. This study aimed to identify the medical causes of sickness absenteeism among adults in Qatar, using a standardized classification scheme.

**Methods:**

A cross-sectional analysis was conducted using data from Electronic Health Records of individuals aged 18 years or older during 2019. The study included all clinical encounters resulting in sick leave. Data were restructured to analyze individuals with sick leaves as the primary unit. A standardized classification scheme for sick leave causes was used. The study reported the relative frequency of reasons for seeking sickness leave and calculated the age and gender specific sick leave incidence rates and the reason-specific rates

**Results:**

During the study period, 387,496 clinical encounters resulting in sick leave were identified, involving 87,738 unique individuals having at least one sick leave. A higher prevalence of sick leave was among younger adults (<25 years) and females, with females being 5.4× more likely to have at least one sick leave per year compared to males. The leading causes of sick leave included cold, cough, flu—influenza (159.9/10,000), and gastrointestinal problems (93.8/10,000). Gender differences were noted, with males experiencing more injuries and infectious diseases, while females reported more genitourinary disorders, headaches, cardiac-related problems, and blood disorders.

**Conclusions:**

This study provides valuable insights into sickness absenteeism in Qatar, highlighting theprimary medical causes. The findings can inform the development of targeted interventions and support systems for a healthier workforce.

## INTRODUCTION

Sickness absenteeism is a phenomenon characterized by absence from work due to illness or injury.^1^ Absenteeism resulting from diverse health conditions serves as a complex indicator of the health status, well-being, and work capacity of employees. Consequently, it is a critical global public health concern.^2,3^ Additionally, evidence showed that being absent due to sickness is a predictive marker for all-cause mortality.^4^ Sickness absence can have unfavorable consequences for the employee, employer, and society. In the realm of employment, absenteeism constitutes a crucial point where the absence of a professional disrupts the workflow, leading to adverse consequences on production. This results in increased costs and places an additional burden on other workers.^5^ Furthermore, the absence of employees from work can shape patterns of diseases and behaviors related to illness within working populations. For instance, providing paid sick leave from the first day of illness can encourage workers to stay at home, thereby preventing the spread of infections.^6^

Understanding sick leave is a crucial issue for workers and their employers. Identifying the reasons and factors underlying sick leave could assist decision-makers in establishing effective prevention policies. This, in turn, could enhance the well-being of workers and concurrently decrease expenses for employers.^7^ Sickness absenteeism represents a complex interplay of factors that extend beyond the immediate health concerns of individuals. Factors at the individual level that have been demonstrated to increase the risk of sickness absence encompass health status, personal attributes (such as age and gender), psychosocial determinants (including personality traits and coping mechanisms), as well as organizational and work-related elements like physical aspects (such as ergonomic factors), psychosocial factors (like job strain and social support), and work schedules.^8–14^ Having a track record of prolonged or recurring sickness absence was found to be associated with increased likelihood of future absences.^15^ In a few cases, sickness absence is not a good or a reliable indicator for morbidity.^16^ Literature indicated that a portion of work nonattendance is more related to factors other than sickness. It suggests that taking sick leave can be a voluntary action influenced by various factors, including the collective attitude towards work and the employee’s satisfaction with their job.^17^

In numerous countries, including Qatar, the certification of sick leave is typically carried out by general practitioners or family physicians in primary healthcare settings.^18^ The process of sickness certification is complex, requiring numerous assessments and assumptions related to the patient’s illness, the credibility of their complaints, their ability to perform workplace tasks, and prognostic estimates regarding the anticipated time for the patient’s return to work.^3,19,20^ Employment regulation in Qatar is governed by Law No. 14 of 2004 (Labor Law). Under this law, an employee is entitled to have paid sick leave for each year of service. However, an employee will only be eligible for sick pay if a physician approved by the employer produces a medical certificate.^21^

Various studies and reviews highlighted diverse common medical reasons for sick leaves, with a predominant consensus indicating that musculoskeletal, respiratory, and mental diseases or symptoms are among the most frequently reported causes.^22–25^ Understanding the root medical causes of sick leaves holds significant importance, encompassing benefits for individual well-being, broader societal health, and economic considerations. Exploring the contributing factors to sickness absenteeism not only addresses the immediate concerns of affected individuals but also enables the development of targeted interventions and support systems. This knowledge proves crucial for organizations aiming to cultivate healthier and more productive work environments. Additionally, at the societal level, such insights inform public health strategies, aiding in the prevention and management of illnesses that contribute to absenteeism. The focus of our study is to explore the medical causes of sick leaves within the specific context of Qatar, offering valuable insights for healthcare professionals and policymakers. To date, and after an extensive literature review, we could not find any study in the Middle East, particularly in Qatar, that explores the causes and incidence of sick leaves in a national cohort. Moreover, there is a scarcity of studies employing systematic classifications for the medical reasons behind sickness absenteeism, making it challenging to facilitate comparisons with other studies. The current study aimed to fill this gap by conducting a thorough analysis to identify the medical causes of sickness absenteeism using a systematic classification in alignment with the internationally recognized International Classification of Diseases (ICD) scheme.^26^ The study will review all sick leaves documented by the Primary Health Care Corporation (PHCC)—the primary healthcare provider in Qatar—offering a unique perspective on the health-related factors that contribute to sick leave in this specific context.

## METHODS

### Study design

Cross-sectional study for the calendar year 2019 (the period from January 1, 2019, to December 31, 2019). This was the most recent year just before the COVID-19 pandemic, which introduced major changes to the health system and sick leaves.

### Study setting

The Electronic Health Records (EHR) maintained by the PHCC cover the 1-year study period from January 1 to December 31, 2019. PHCC is the largest public facility providing primary healthcare in Qatar. At the time of the study, PHCC operated 27 health centers distributed across the country and served a population of more than 1.5 million people. The services provided were accessible and largely subsidized in cost.

### Study population

The targeted population comprised all the clinical encounters of adults aged 18 years and above in any of the 27 health centers operated by PHCC with a sick leave as an outcome during the designated study period. No sampling procedure was followed, and all the valid cases were analyzed. A total of 387,496 clinical encounters ending in sick leave were identified during the study period. These resulted in a total of 87,738 unique individuals who obtained at least one sick leave during the study year.

### Study variables

These include gender, age in years, reported reason recorded by the physician, length of sick leave in days, and date of sick leave.

### Data access and cleaning methods

The Business Health Intelligence (BHI) department of PHCC was responsible for data extraction using custom-designed database filters. The experts in BHI follow rigorous work processes, and the filters developed are cross-checked and tested by a second team before being shared.

Preliminary analysis of de-identified data extracted by the BHI showed 11,086 unique reasons reported on the sick leave forms included in the current study. These were recorded by the study team using 25 distinctive codes denoting specific health condition categories defined by the Sickness Absence Recording Tool (SART).

### Classifying the reported reasons for sick leaves

A standardized classification scheme for sick leave causes was developed by the Institute of Occupational Medicine as part of the SART project in collaboration with the Health and Safety Executive UK in 2002 to 2004. This scheme comprises two hierarchical levels of coding, with 23 categories (in addition to another two nonspecific categories) at level 1 specifying the main body system affected by the illness or ailment, as shown in Table 1.^26^

The SART project aimed to create a simple, standardized SART that could be used by organizations that currently have no absence management and recording system in place. The tool can be used to investigate the status of sickness absence recording and assist employers in managing sickness absence. The classification scheme is designed to allow employers to easily classify in a standardized way the reasons for sickness absence provided by employees, whether conveyed by verbal reports, self-certificates, or medical certificates issued by a healthcare professional. The scheme has been devised to be broadly compatible with the internationally recognized ICD scheme at the top level, so as to enable the comparison of rates in the future, particularly with information gathered at a local level and used to influence planning of health and related services. The SART was evaluated and tested by the University of Glasgow.^27^

The raw data provided by the BHI contained a total of 387,496 sick leave encounters during the 1-year study period. The primary sampling unit was the sick leave incident. This data set was used to report the frequency distribution of reported causes for sick leaves. Furthermore, since an individual can have more than one sick leave encounter during a year, the same data were restructured to obtain individuals acquiring sick leaves as the primary sampling unit. A total of 87,738 unique individuals obtained at least one sick leave during the study year. The second database was used to calculate incidence rates by referring to the population at risk of seeking a sick leave (adults aged 18 years and above). The population at risk estimates obtained may suffer from an overestimation bias due to the fact that some elderly individuals are not working. This bias is assumed to be small in size (Qatar population is heavily skewed towards young ages, with those aged 55 years and above constituting only 3.6% of the total population of adults in Qatar).

The REporting of studies Conducted using Observational Routinely-collected health Data (RECORD) Statement, which is an extension of the STROBE statement checklist (International Collaborative Initiative for Strengthening the Reporting of Observational Studies in Epidemiology) specially designed to assure the quality of reporting of secondary data analysis and was followed during analysis and writing of the research paper.

### Statistical analysis

The statistical package of social sciences (IBM; SPSS, version 28) was used to perform statistical analysis. The relative frequency of level 1 SART classified reasons for seeking a sickness certificate in clients registered with the PHCC was reported. The age- and gender-specific sick leave incidence rates and the reason-specific rates were calculated by referring to the quarterly population estimates reported by the Statistics and Planning Authority of Qatar.28 The 2019 population estimates were adjusted to remove the single male worker population, which receives its health services from the Red Crescent of Qatar (Table 2).

### Ethical approvals

This study was performed in line with the principles of the Declaration of Helsinki. The study protocol was reviewed and approved by the PHCC Research Subcommittee (PHCC/DCR/2020/06/054).

## RESULTS

The majority of participants fell within the 25 to 44 years age range (64.7%). Qatari nationals accounted for 45.9% of the total participants. Moreover, females slightly outnumbered males, comprising 54.9% compared to 45.1% (Table 3).

As shown in Table 4, compared to the oldest age (55+ years), the youngest age group (18–24 years) was associated with a 50% increase in the risk of having at least one sick leave per year. The incidence rate of obtaining at least one sick leave per year ranged between as low as 5% for those aged 35 to 44 years to as high as 8.6% for those 18 to 24 years old. Females are 5.4× more likely than males to have at least one sick leave per year. The incidence rate of reported reasons for sick leave among a total at-risk population of slightly more than one and a half million working adult forces in Qatar was reported in Table 5. The top five reasons were: cold, cough, flu—influenza (159.9/10,000), gastrointestinal problems (93.8/10,000), ear, nose, and throat (62.8/10,000), other musculoskeletal problems (40.9/10,000), and back problems (37.9/10,000).

Gastrointestinal problems and pregnancy-related disorders were more commonly reported as reasons for sick leave among younger individuals. In contrast, the other musculoskeletal problems (excluding back problems), and the heart, cardiac, and circulatory problems were notably less frequent among young ages (Table 6). The genitourinary and gynecological disorders (excluding pregnancy- related disorders) were notably less frequent among older ages. Nevertheless, among the same older people, the eye problems, chest and respiratory problems—(exclude nose and throat problems, cold, cough, flu), endocrine, nutritional, and metabolic diseases (e.g., diabetes, thyroid, metabolic problems), and asthma were notably more frequent when compared to younger ages (Table 6).

As shown in Table 7, cold, cough, flu—influenza, injury, fracture, and infectious diseases were the reasons more commonly reported among males. In contrast, the genitourinary and gynecological disorders (excluding pregnancy-related disorders), headache/migraine, heart, cardiac, and circulatory problems, blood disorders (e.g., anemia), and (obviously) pregnancy-related disorders were more frequently reported as reasons for sick leave among females.

## DISCUSSION

Sickness absenteeism is intricately shaped by a multifaceted interplay of factors that extend beyond the immediate health concerns of individuals. Notably, physicians exhibit substantial variation in their treatment approaches, both across different geographic regions and within the same locale, even when the health status of the patient remains constant.^29^ This variance arises from the fact that each physician harbors a unique set of preferences and beliefs concerning the necessity and effectiveness of different treatments. This diversity in medical practices has direct implications for sick leave certification as well. The findings of this study provide a comprehensive understanding of sickness absenteeism in Qatar.

The results of the current study showed that younger adult individuals (<25 years of age) are much more likely to obtain sick leaves compared to their older counterparts. This pattern aligns with findings from various studies and reports.^24,25,30–35^ For instance, a study in Finland focusing on physically demanding occupations, such as construction and maintenance jobs, demonstrated that although self-reported health problems increased with age, the prevalence of absenteeism was notably higher in the 18 to 30 years age group, where 71% had been absent from work, compared to 53% in the 55 to 61 years age group.^35^ This suggests that younger workers tend to stay away from work more frequently due to minor health issues, emphasizing that sickness absenteeism is influenced by factors beyond medical conditions alone. These factors include perceived ability to work, cultural variations in the importance of being assigned to work, and differential perceptions of the potential consequences of absence from work. Furthermore, a study in a neighboring country, Saudi Arabia, observed a significantly higher rate of sick leave among younger employees (40 years old or less) compared to those aged 41 years or older.^24^ Other explanations for this negative relationship between age and sick absenteeism, with older workers having fewer spells of absence, include older workers having a greater need for regularity,^36^ and having greater family and financial responsibilities that would deter them from staying at home as a result of a minor illness.^37^ However, this inverse association is not uniformly observed. Few studies showed a contrasting trend, identifying a positive relationship between age and sickness absence.^38,39^

Our study found a notable gender disparity, indicating that females are significantly more prone to experiencing at least one sick leave per year compared to males. This is consistent with findings from other studies.^38,40–43^ Some researchers, such as Laaksonen et al., suggest that this difference is particularly evident for short spells, though our study did not specifically assess this aspect.^43^ Another study highlighted that in comparison to men, a higher percentage of women both require and take leave.^41^ A possible explanation for these gender differences includes health issues related to pregnancy and childbirth. Furthermore, women are more likely to grapple with chronic health conditions like asthma, migraine, and depression, which may necessitate more frequent sick days to manage symptoms. However, Østby et al. found that despite adjusting for a large number of factors, including health, job, and family dynamics, a considerable portion of the gender gap in sickness absence remains unexplained.^40^ Another contributing factor is the likelihood of women serving as primary caregivers for children or elderly relatives, introducing additional stressors that may prompt the need for sick days to address family emergencies. Additionally, societal gender stereotypes may shape attitudes toward sickness absence, portraying traditional women as weak and dependent and traditional men as competitive and independent, possibly rendering women more susceptible to sickness absence.^44^ Furthermore, women may be more likely to take sick days because they feel less able to say no to their employers. On the other hand, few studies found no relationship between gender and sick leaves, as there was no difference in the sick leave rate between males and females.^24^

The lack of a universally standardized classification for sick leave reasons complicates the comparability between different studies and may result in misinterpretations. Therefore, when interpreting our results, it is crucial to consider the SART classification methodology employed in this study, which aligns with the ICD. Therefore, it is imperative to consider the following points: first, in the SART classification, the category of “chest and respiratory problems” excludes cold, cough, flu—influenza, and ear, nose, throat problems (encompassing laryngitis, pharyngitis, blocked nose, etc). These conditions are regarded as part of respiratory problems or upper respiratory conditions in numerous other studies. Additionally, SART treats asthma as a distinct category separated from other chest and respiratory problems. Second, this classification distinguishes back problems from other musculoskeletal problems, a departure from the approach in many other studies. Our research findings showed that the top five reasons for sick leaves in Qatar are cold, cough, flu—influenza; gastrointestinal problems; ear, nose, throat problems; other musculoskeletal problems; and back problems, which align with broader patterns observed in other studies, where one or more of the mentioned causes consistently emerges as one of the top three reasons for sick leaves in their respective analyses.^23–25,38,45–51^

In our study, the prevalence of psychiatric-related reasons for sick leaves was relatively low across gender and age categories, contrasting with other studies and reports in which psychiatric causes were identified among the most frequent.^25,38,47–50^ This discrepancy may reflect unique behavioral patterns, particularly those influenced by social culture and stigma-related factors in our region. Despite increasing acceptance of mental health disorders in the Arab Gulf region due to awareness campaigns and public health education, sociocultural challenges, including financial barriers, shame, and stigma, continue to significantly impact mental health perceptions.^52^ Traditionally, stigma acts as a barrier for individuals with mental health issues in seeking proper care. As a result, mental illness often goes unreported as a cause of sickness absenteeism.^53^ A qualitative study in Qatar revealed that participants expressed fear of potential repercussions on their employment if others were aware of their mental health problems.^54^ Additionally, limited awareness and understanding of mental health conditions, extending even to healthcare providers responsible for issuing absence certifications, may contribute to this finding. Moreover, mental health issues might manifest with physical symptoms, which might lead to their misclassification and a failure to properly recognize them as mental health reasons for sick leaves. Furthermore, the workplace environment plays a crucial role, as a negative and nonsuppurative workplace culture can prevent individuals from openly discussing and reporting their mental health concerns.

The leading causes of sick leaves are consistent across genders, which is aligned with most of the literature^23,55^; however, the primary distinctions are as follows. Males exhibited a higher prevalence of infectious diseases, injuries, and fractures, likely due to their engagement in physically demanding occupations, leading to increased risks of accidents. Moreover, their engagement in fieldwork, travel- centric roles, and participation in riskier health sector specialties contribute to a heightened susceptibility to infectious diseases. In contrast, females reported higher rates of genitourinary and gynecological disorders, headache/migraine, cardiac issues, blood disorders, and pregnancy-related disorders. While limited studies have documented absence reasons by gender, those available have shown similar general patterns.^23,55^ One study in France indicated that females had more respiratory, genitourinary, and cardiovascular-related sick leaves, akin to our findings, but fewer accidents and mental health-related leaves, contradicting ours.^23^ Furthermore, a London-based study identified respiratory and gastrointestinal problems as the top two reasons in both genders, and it observed that women experience more sick leaves due to headache, migraine, genitourinary, cardiovascular, and respiratory issues, mirroring our results.^55^ However, it reported almost equal percentages of injury-related leaves for both genders, inconsistent with our findings.^55^ It has been proposed that the most significant gender disparity in disease categories, for which both men and women seek medical care, lies in conditions associated with mild morbidity and those where there is a greater discretion in defining illness or determining the need for care. Accordingly, these distinctions might arise from variations in the perception, evaluation, and response to symptoms.^56^ These differences can be attributed to anatomical disparities between genders. Additionally, females often perceive symptoms like headaches, migraines, and cardiac issues (chest pain and shortness of breath) as more serious and warrant a sick leave. Another explanation is that the impact of the work/ family interface on health is more pronounced for women, resulting in women experiencing more minor subjective illnesses. Alternatively, the work/family interface itself might be the primary cause of absence, but a subjective medical reason could be provided, possibly perceived as more acceptable. This could be consistent with women’s greater reporting of absence for headache and migraine. The rise in sick leaves attributed to cardiovascular-related conditions or symptoms is probably linked to the higher reporting of angina by women, especially in screening examinations.^57^ Additionally, the increased utilization of health services by women implies a higher likelihood of screening, potentially leading to the identification and treatment of CVD-related conditions such as hypertension.^55^

### Strengths and limitations

This study exhibits notable strengths stemming from its utilization of a credible source of information, which is the EHR sourced from the PHCC, the main primary healthcare provider in Qatar. It is distributed across 27 centers spanning the entire geographical area of the country. Encompassing a significant portion of the working population, except for single male workers. Rigorous data extraction and cleaning procedures, complemented by the use of the standardized SART classification scheme, contribute to heightened reliability and comparability of our findings. Pioneering in the Middle East, this study is one of the rare instances utilizing a standardized tool specifically tailored for the classification of sick leaves. The precision in estimating relative risk for sick leave positions makes this research a foundational resource for future investigations into sick leave patterns in Qatar. Nevertheless, the study grapples with certain limitations that warrant acknowledgment. Dependency on healthcare records may result in an oversight of sick leaves managed outside the governmental healthcare system or those involving secondary care for emergency and critical situations. The retrospective nature of the study, lacking primary data collection, restricts the exploration of intersecting factors influencing absenteeism, such as socio- economic status, job characteristics, and cultural determinants. Additionally, the study falls short in scrutinizing the legitimacy of sick leaves, as it lacks primary data from PHCC doctors regarding their opinions. Determinants and predictors of sick leave duration, as well as the frequency of sick leaves per individual, remain unexplored facets in this investigation.

## CONCLUSIONS

In conclusion, this study provides valuable insights into the complexities of sickness absenteeism in Qatar, offering a detailed analysis of demographic associations and primary causes. The comprehensive findings lay a solid foundation for targeted interventions aimed at cultivating a healthier and more resilient workforce. As Qatar undergoes economic and demographic transformations, the study’s results become a vital resource for shaping evidence-based policies in occupational health and well-being and tailoring initiatives based on age and gender-specific considerations. Workplace health programs should focus on preventive measures for common illnesses, ergonomic improvements, and flexible work policies. Gender-sensitive initiatives, including reproductive health support and childcare assistance, can help mitigate absenteeism. Expanding occupational health services, vaccination campaigns, and data-driven policy adjustments will further enhance workforce well-being.

### Authors’ contribution

**Sami Abdeen**: Conceptualization, Methodology, Writing—original draft preparation, Writing— review and editing. **Ahmed Sameer Alnuaimi**: Conceptualization, Methodology, Formal analysis, Data curation, Writing—original draft preparation, Writing—review and editing, Supervision. **Muna Abed Alah**: Conceptualization, Methodology, Writing—original draft preparation, Writing— review and editing. **Salwan Al-Kubaisi**: Conceptualization, Methodology, Writing—review and editing. **Iheb Bougmiza**: Writing—review and editing, Supervision. All authors contributed to the study conception and design. All authors read and approved the final manuscript.

### Funding

This research did not receive any specific grant from funding agencies in the public, commercial, or not- for-profit sectors.

### Conflict of interest

The authors have no competing interests to declare that are relevant to the content of this article.

### Data availability statement

Data will be available upon reasonable request from the corresponding author.

## Figures and Tables

**Table 1.** Level 1 (top level) classification scheme of the Sickness Absence Recording Tool (SART).

Cold, cough, flu—influenza Gastrointestinal problems (e.g., abdominal pain, gastroenteritis, vomiting, diarrhea)—exclude dental and oral problems Ear, nose, throat Other musculoskeletal problems—exclude back problems and include neck problems Genitourinary and gynecological disorders—exclude pregnancy-related disorders Back problems Dental and oral problems Headache/migraine Injury, fracture Skin disorders Eye problems Heart, cardiac, and circulatory problems Chest and respiratory problems—exclude nose and throat problems, asthma, cold, cough, flu Endocrine, nutritional, and metabolic diseases (e.g., diabetes, thyroid, metabolic problems) Infectious diseases Pregnancy-related disorders Asthma Blood disorders (e.g., anemia) Burns, poisoning, frostbite, hypothermia Anxiety/stress/depression/other psychiatric illnesses Nervous system disorders—exclude headache/migraine Benign and malignant tumors, cancers Substance abuse—including alcoholism and drug dependence Other known causes (nec)—not elsewhere classified in this scheme Unknown causes/not specified

**Table 2.** Adjusted population estimates for the year 2019.

Age group (y)	Qatari		Expatriates	
	Male	Female	Male	Female
18–24	13,268	6,243	136,958	33,714
25–34	22,281	15,658	485,187	94,145
35–44	16,066	10,666	367,886	86,377
45–54	12,676	5,786	166,244	27,258
55+	4,874	1,017	43,191	7,024
Total	69,165	39,370	1,199,466	248,518

**Table 3.** Frequency distribution of the study group with at least one sick leave during the study year by age, gender, and nationality.

Characteristics		*N*	%
	18–24	16,423	18.7
	25–34	32,559	37.1
Age group (y)	35–44	24,206	27.6
	45–54	11,289	12.9
	55+	3,261	3.7
Nationality groups	Expats	47,457	54.1
	Qatari nationality	40,281	45.9
Gender	Female	48,142	54.9
	Male	39,596	45.1
Total		87,738	100.0

**Table 4.** The incidence rate of having at least one sick leave per year from the total workforce.

Characteristics		*N* at risk	*N* sick leave	Rate (/10,000)	Relative risk
	18–24	190,183	16,423	863.5	1.5
	25–34	617,271	32,559	527.5	0.9
Age group (y)	35–44	480,995	24,206	503.2	0.9
	45–54	211,964	11,289	532.6	0.9
	55+	56,106	3,261	581.2	Ref
Gender	Female	287,888	48,142	1,672.2	5.4
	Male	1,268,631	39,596	312.1	Ref
Total		1,556,519	87,738	563.7	

**Table 5.** The reason specific incidence rate of having at least one sick leave per year from the total workforce.

Reported reason for sick leave	*N* sick leave	Rate (/10,000)*
Cold, cough, flu—influenza	24,894	159.9
Gastrointestinal problems (e.g., abdominal pain, gastroenteritis, vomiting, diarrhea)—exclude dental and oral problems	14,599	93.8
Ear, nose, throat	9,770	62.8
Other musculoskeletal problems—exclude back problems and include neck problems	6,368	40.9
Back problems	5,895	37.9
Genitourinary and gynecological disorders—exclude pregnancy-related disorders	5,048	32.4
Dental and oral problems	4,267	27.4
Injury, fracture	3,508	22.5
Other known causes (nec)—not elsewhere classified in this scheme	3,394	21.8
Headache/migraine	3,138	20.2
Heart, cardiac, and circulatory problems	3,006	19.3
Eye problems	2,574	16.5
Skin disorders	2,485	16
Chest and respiratory problems—exclude nose and throat problems, asthma, cold, cough, flu	2,424	15.6
Endocrine, nutritional, and metabolic diseases (e.g., diabetes, thyroid, metabolic problems)	1,774	11.4
Pregnancy-related disorders	1,765	11.3
Infectious diseases	1,633	10.5
Asthma	870	5.6
Blood disorders (e.g., anemia)	486	3.1
Burns, poisoning, frostbite, hypothermia	394	2.5
Nervous system disorders—exclude headache/migraine	324	2.1
Anxiety/stress/depression/other psychiatric illnesses	258	1.7
Benign and malignant tumors, cancers	111	0.7
Unknown causes/not specified	34	0.2
Substance abuse—includes alcoholism and drug dependence	16	0.1

*N for the total at-risk population = 1,556,519.

**Table 6.** The relative frequency of selected reasons for sick leave by age group.

Reported reason for sick leave	Age group (y)									
	18–24 (total *N* = 1,642)		25–34 (total *N* = 32,559		35–44 (total *N* = 24,206)		45–54 (total *N* = 11,289)		55+ (total *N* = 3,261)	
	*N*	%	*N*	%	*N*	%	*N*	%	*N*	%
Cold, cough, flu—influenza	4,664	28.4	9,546	29.3	6,821	28.2	2,978	26.4	885	27.1
Gastrointestinal problems (e.g., abdominal pain, gastroenteritis, vomiting, diarrhea)—exclude dental and oral problems	4,331	26.4	5,465	16.8	3,183	13.1	1,293	11.5	327	10
Ear, nose, throat	1,485	9	3,777	11.6	2,950	12.2	1,240	11	318	9.8
Other musculoskeletal problems—exclude back problems and include neck problems	684	4.2	1,979	6.1	2,085	8.6	1,284	11.4	336	10.3
Back problems	622	3.8	2,160	6.6	1,870	7.7	989	8.8	254	7.8
Genitourinary and gynecological disorders—exclude pregnancy- related disorders	1,289	7.8	1,976	6.1	1,205	5	491	4.3	87	2.7
Dental and oral problems	657	4	1,604	4.9	1,283	5.3	568	5	155	4.8
Injury, fracture	691	4.2	1,367	4.2	922	3.8	406	3.6	122	3.7
Other known causes (nec)—not elsewhere classified in this scheme	579	3.5	1,303	4	922	3.8	483	4.3	107	3.3
Headache/migraine	568	3.5	1,091	3.4	961	4	444	3.9	74	2.3
Heart, cardiac, and circulatory problems	333	2	862	2.6	976	4	614	5.4	221	6.8
Eye problems	349	2.1	842	2.6	780	3.2	433	3.8	170	5.2
Skin disorders	467	2.8	902	2.8	693	2.9	322	2.9	101	3.1
Chest and respiratory problems—exclude nose and throat problems, asthma, cold, cough, flu	269	1.6	827	2.5	746	3.1	394	3.5	188	5.8
Endocrine, nutritional, and metabolic diseases (e.g., diabetes, thyroid, metabolic problems)	163	1	416	1.3	510	2.1	459	4.1	226	6.9
Pregnancy-related disorders	176	1.1	1,173	3.6	411	1.7	5	<0.1	0	0
Infectious diseases	332	2	640	2	421	1.7	183	1.6	57	1.7
Asthma	144	0.9	279	0.9	236	1	150	1.3	61	1.9
Blood disorders (e.g., anemia)	78	0.5	174	0.5	143	0.6	83	0.7	8	0.2
Burns, poisoning, frostbite, hypothermia	65	0.4	159	0.5	117	0.5	41	0.4	12	0.4
Nervous system disorders—exclude headache/migraine	49	0.3	108	0.3	104	0.4	46	0.4	17	0.5
Anxiety/stress/depression/other psychiatric illnesses	50	0.3	93	0.3	71	0.3	33	0.3	11	0.3
Benign and malignant tumors, cancers	11	0.1	27	0.1	38	0.2	27	0.2	8	0.2
Unknown causes/not specified	3	<0.1	11	<0.1	12	<0.1	7	0.1	1	<0.1
Substance abuse—includes alcoholism and drug dependence	1	<0.1	4	<0.1	7	<0.1	4	<0.1	0	0

**Table 7.** The relative frequency of selected reasons for sick leave by gender.

Reported reason for sick leave	Gender			
	Female (total *N* = 48,142)		Male (total *N* = 39,596)	
	*N*	%	*N*	%
Cold, cough, flu—influenza	12,588	26.1	12,306	31.1
Gastrointestinal problems (e.g., abdominal pain, gastroenteritis, vomiting, diarrhea)—exclude dental and oral problems	8,065	16.8	6,534	16.5
Ear, nose, throat	5,315	11	4,455	11.3
Other musculoskeletal problems—exclude back problems and include neck problems	3,702	7.7	2,666	6.7
Back problems	3,189	6.6	2,706	6.8
Genitourinary and gynecological disorders—exclude pregnancy-related disorders	4,425	9.2	623	1.6
Dental and oral problems	2,223	4.6	2,044	5.2
Injury, fracture	1,250	2.6	2,258	5.7
Other known causes (nec)—not elsewhere classified in this scheme	2,121	4.4	1,273	3.2
Headache/migraine	2,338	4.9	800	2
Heart, cardiac, and circulatory problems	1,963	4.1	1,043	2.6
Eye problems	1,378	2.9	1,196	3
Skin disorders	1,253	2.6	1,232	3.1
Chest and respiratory problems—exclude nose and throat problems, asthma, cold, cough, flu	1,065	2.2	1,359	3.4
Endocrine, nutritional, and metabolic diseases (e.g., diabetes, thyroid, metabolic problems)	1,008	2.1	766	1.9
Pregnancy-related disorders	1,762	3.7	0	0
Infectious diseases	654	1.4	979	2.5
Asthma	444	0.9	426	1.1
Blood disorders (e.g., anemia)	465	1	21	0.1
Burns, poisoning, frostbite, hypothermia	220	0.5	174	0.4
Nervous system disorders—exclude headache/migraine	186	0.4	138	0.3
Anxiety/stress/depression/other psychiatric illnesses	154	0.3	104	0.3
Benign and malignant tumors, cancers	72	0.1	39	0.1
Unknown causes/not specified	24	<0.1	10	<0.1
Substance abuse—includes alcoholism and drug dependence	3	<0.1	13	<0.1
